# 

**Published:** 1919-08-09

**Authors:** 


### THE COLLEGE OF NURSING.

### Its Coat of Arms and Badge; An Attractive Competition.

IT is proposed to apply for a Coat of Arms and Badge for the College of Nursing, and it is thought that members of the College and those interested in it may desire to express their views as to the emblems and mottoes which might most suitably be adopted as indicative of the work and objects of the College.

In order to ascertain these views the Council has accepted, with grateful thanks, the offer of THE HOSPITAL of a prize of £10 for the best design for a Coat of Arms, and the College offers a first prize of £7 10s., and a second prize of £2 10s., for the best design for a Badge, with a view to these being submitted, if thought suitable, to the proper authorities. Competitors are invited to send in their designs in a covered envelope marked "Coat of Arms" and "Badge" respectively.

Simplicity in design should be aimed at in both, which should be suitable to be borne on either nurses' uniforms or on the property of the College generally.

The designs must be received on or before September 30, and addressed to the Secretary, the College of Nursing, Ltd., 7 Henrietta Street, Cavendish Square, W. 1.

### PASSING EVENTS AND TOPICS.

### The Wellcome Historical Medical Museum.

This museum will be closed for cleaning and redecoration from August 9 until the end of September.

### Lady Welfare Superintendents.

Lady Welfare Superintendents are about to be appointed by the Co-operative Wholesale Society, Ltd., for their various productive factories.

### Grant of Charter of Incorporation.

The King has granted a Charter of Incorporation to the Royal Hospital and Home for Incurables, Putney, of which institution His Majesty is patron.

### Fellowship for Malignant Diseases Research.

Mr. John Duff, M.D., who left £9,752, gave all his property in trust for his two sisters for life and, on the death of the survivor, "to the Glasgow University to form a fellowship for the elucidation of those malignant diseases that kill so many people."

### Croix de Guerre for Englishwomen.

Miss A. M. Dobson and Miss E. de S. Preedy have been decorated with the Croix de Guerre for valour under bombardment near the front, in addition to the Médaille de la Reconnaissance Français for devotion to duty. They served for three years under the French Red Cross.

### New Sanatorium for Essex.

The ninth sanatorium which has been established in Essex under the Insurance Act of 1911 was opened on Friday, July 18, at High Beech, by Mr. Andrew Johnston, J.P. It is to be used for children suffering from non-pulmonary tuberculosis, and is named "The Suntrip."

### The Milk Supply.

The National Association for the Prevention of Infant Mortality has called the attention of the Government to the urgent necessity for reconstruction of the milk supply throughout the country, it being asserted that the quantity and quality of the supply at present is in a deplorable state. It is urged that the earnest attention to the problem is vital to the babies and children of to-day.

### Motor Transport War Work.

The Bishop of Kensington has unveiled a memorial tablet in St. James' Church, Piccadilly, to the memory of the men of the Motor Transport Department, British Red Cross Society, who have fallen during the war. Seven hundred members of the motor transport met at dinner at the Connaught Rooms—a reunion of those who had served at home and abroad. In the course of the speeches it was mentioned that of the 9,700,000 cases driven, not one man had been injured by the fault of the drivers.

### The Test of Bee-Stings.

Speaking at a lecture on Wednesday, July 30, at the British Science Exhibition, Central Hall, Westminster, Mr. W. T. Reid, late President of the British Bee-Keeping Association, said he had found the poison of the bee an almost infallible test as to whether a person would be likely to succumb under an anæsthetic. In a person suffering from a form of nervous weakness, a bee-sting on the hand would cause a large swelling and affect the glands of the body. That person should not be operated upon. It would be interesting to investigate such a theory further. The beehives in the hospital garden might fulfil a more useful purpose than supplying the honey for the patients' tea.

---

A new hospital is to be erected at Retford as a war memorial.

Lincoln is considering a scheme to provide a maternity hospital at Garmston House, Newland.

Cottage hospitals are to be built at Dudley (Northumberland) and Earlestown (Lancashire).

Nearly £4,000 has been promised towards the erection of a cottage hospital at Earlestown.

Steps are being taken in Monmouthshire to build three isolation hospitals, at an estimated cost of £100,695.

### Gifts and Bequests.

Mr. W. H. Spurr, of Addington, Yorks, left £400 each to the Saltaire Hospital and the Bradford Eye and Ear Hospital.

Mr. Dugald Munn, of Whitehall Court, S.W., has left £500 to the Victoria Hospital, Rothesay, and £100 to the London Hospital.

Under the will of the late Mr. T. G. Langham, of Leicester, a sum of £3,000 each is left to the National Lifeboat Institution and the Butchers' Charitable Institution.

The Harrogate Infirmary have just recently received a cheque for £290, the proceeds of a series of successful entertainments organised by the Associated Clubs of Harrogate. The Hon. Treasurer of the infirmary expressed on behalf of the committee their high appreciation of this splendid effort, and thanked all the hard-working organisers and helpers in the successful scheme.

Lady Haliburton, of Lowndes Square, S.W., has left the residue of her property to the Metropolitan Convalescent Institution for the ward to be called "The Lee Schuster Ward."

Sir Henry Webb, Bart., has given 1,000 guineas as part of the residuary estate of the late Lady Webb to endow a second bed at the Prince of Wales' Hospital for Limbless Sailors and Soldiers, Cardiff. These beds are in memory of Lieutenant Basil Webb, who was killed in action in 1917.

Mr. Henry Rogers, of Dorset Square, W., whose estate is valued at £85,473, after making provision for his sister and others, left his property in trust for three nieces for life, and on the death of the survivor of them to the Dental Hospital, Leicester Square, St. Bartholomew's Hospital, Dr. Barnardo's Homes, and the Church Army.

### The College of Nursing (Limited by Guarantee).

SCOTTISH BOARD.

The following have been elected to the fifteen vacancies on the Scotttish Board. The election was carried out by postal ballot:—Sir James O. Affleck, M.D., LL.D., F.R.C.P.Ed.; Miss Elizabeth Graham, matron, Scottish Association of Trained Nurses; Miss Margaret White, General Superintendent, Scottish Branch, Q.V.J.I.N.; Lt.-Col. Sir Joseph Fayrer, Bart., C.B.E., F.R.C.S.; James Hodsdon, Esq., F.R.C.S.Ed.; Miss A. M. Milligan, matron; Miss Elizabeth Cumming, matron; Miss Jane Thomas, matron; Miss Nellie Cathcart, trained nurse; Miss Annie Matheson, night superintendent of nurses; John M'Cubbin Johnston, Esq., M.A., M.D.; Miss Agnes Lindsay, matron; Miss Margaret Bell, Queen's Nurse; Miss Janet Rodger, matron; Miss Esther Keay, trained nurse.

The remaining members of the Scottish Board are:—*Members appointed under the Constitution*:—C. B. Ker, Esq., M.D.; David Wallace, Esq., F.R.C.S.E.; Ebenezer Duncan, Esq., M.D.; Mrs. George Kerr; J. Maxton Thom, Esq., M.D.; Miss Edmonson, R.R.C. (also an *ex-officio* member); John W. Fleming, Esq.; Miss Gregory Smith, R.R.C.

*Ex-Officio Members*:—Miss Gill, R.R.C.; Professor Glaister, M.D.; D. J. Mackintosh, Esq., C.B., M.V.O., LL.D.; Miss Melrose, R.R.C.; Professor Ritchie, M.D., F.R.C.P.

The office of the Scottish Board at 122 George Street, Edinburgh, will be closed during August.

EDINBURGH CENTRE.

At a meeting of members held on Wednesday, July 23, at 29 Castle Terrace, the training-home for district nurses of the Q.V.J.N.I. in Scotland, at which Miss Graham, Chairman of the Executive Committee of the Centre, presided. A paper on "Nominating and Voting at an Election" was read by Miss McKean, one of the nurses at present in the training-home.

Another member read the article on "Propaganda" which appeared in The Hospital of July 19. The interest aroused by both articles was evidenced in the animated discussion which followed, members present resolving to use every effort to further the interests and development of the College.

Between 90 and 100 nurses were present at a garden party given to the members of the Edinburgh Centre of the College of Nursing by Mrs. Turnbull, "The Elms," Whitehouse Loan, on Tuesday, July 29. The weather was lovely, and the games and competitions gave much pleasure and amusement, the prize-winners receiving great applause.

BIRMINGHAM CENTRE.

On Tuesday, July 29, a most interesting lecture was given by Mr. W. Billington, F.R.C.S., on the treatment of abdominal cases before and after operation. Owing to the holidays there was not quite such a good attendance as had been expected, and it is hoped that in future members will make a special effort to attend all lectures arranged by the Centre.

### MATRONS' AND SISTERS' APPOINTMENTS.

Cottage Hospital, Oakdale, Blackwood, Mon.—Miss Hilda C. Evans has been appointed matron. She was trained at King Edward VII.'s Hospital, Cardiff, and at Plaistow Hospital, being later staff nurse in the children's ward and *x*-ray department at the former institution. She has also been staff nurse at the Children's Hospital, Harold Wood, Essex, ambulance nurse for East London districts, and ward sister at the Cottage Hospital, Oakdale. Miss Evans is a member of the College of Nursing.

Coronation Cottage Hospital, Ilkley, Yorks.—Miss Mary Leacy has been appointed matron. She was trained at the London Hospital, and has served with Queen Alexandra's Imperial Military Nursing Service Reserve in France. She holds the certificate of the Central Midwives Board and the Massage Certificate of the London Hospital.

Home of Recovery, Allerton Towers, Woolton, Liverpool.—Miss E. A. Cockburn Hughes, A.R.R.C., has been appointed matron. She was trained at the Royal Infirmary, Liverpool, and has been engaged in district and private nursing abroad and in England. She served with the St. John Ambulance at St. Malo and at the Brigade Hospital, Etaples, and has been matron of the Gostwycke V.A.D. Hospital, Colchester. Miss Hughes holds the certificate of the Central Midwives Board.

Borough of Woolwich Home for Ailing Babies.—Miss J. Raymond has been appointed matron. She was trained at the Queen's Hospital for Children, Hackney Road, and has been home sister at the South-Eastern Hospital for Children, Sydenham.

Lambeth Infirmary, S.E.—Miss M. C. Treharne Jones has been appointed assistant matron. She was trained at the Kent and Canterbury General Hospital, has been staff nurse at the Royal Sussex County Hospital, Brighton, superintendent of nurses at Stow Hill Infirmary, Newport, Mon., matron of a temporary hospital for an epidemic of typhoid, and matron of military hospitals under the Joint War Committee. Miss Treharne Jones holds the certificate of the Central Midwives Board.

Women's Hospital, Nottingham.—Miss Grace Townend has been appointed sister. She was trained at the Royal Infirmary, Manchester, where she was later sister of the gynæcological wards. Miss Townend has also done military and private nursing.

Workhouse Infirmary, Chippenham.—Miss Ethel Amelia Foreman has been appointed head nurse. She was trained at the Parish Infirmary, Shirley Warren, Southampton, where she has since been staff nurse.

Workhouse Infirmary, Great Yarmouth.—Miss Edith Traynor has been appointed night superintendent. She was trained at Newcastle-on-Tyne Infirmary, where she was later ward sister. Miss Traynor has also been a staff nurse in the Territorial Force Nursing Service.

St. Pancras Infirmary (South), Pancras Road, N.W.—Miss D. Boscawen, Miss E. E. Moon, and Miss E. B. Elgar have been appointed sisters. Miss Boscawen and Miss Moon were trained at Westminster Infirmary, Hendon, and both have been staff nurse and sister at the Norfolk War Hospital, Norwich. Miss Elgar was trained at Camberwell Infirmary, where she was later staff nurse and sister, and she has since been sister in Queen Alexandra's Imperial Military Nursing Service Reserve.

Poor-Law Infirmary, Chelmsford.—Miss L. S. Thomas has been appointed superintendent nurse. She was trained at the Greenwich Union Infirmary, and has been superintendent nurse at the Isle of Thanet Union Infirmary, Bridgend and Cowbridge Union Infirmary, Bromsgrove Union Infirmary, and at the Chelmsford Infirmary.

Tunstall.—Miss Jeannie Trueman has been appointed town nurse for Tunstall. She was trained at Wolstanton and Burslem Union Hospital, where she was later night sister. She took her midwifery training at the Queen Victoria Nursing Institute, Northampton.

Carnarvonshire and Anglesey Infirmary, Bangor.—Miss Jane Mortimer has been appointed staff nurse. She was trained at Preston Royal Infirmary, where she later took temporary duties as sister of the out-patient department, the *x*-ray and electrical department, and the V.D. clinic.

Todmorden Union Infirmary.—Miss E. McWaters has been appointed charge nurse at the above infirmary. She was trained at Blackburn Poor-Law Infirmary, later being staff nurse there, and has been charge nurse at Scarborough Poor-Law Infirmary. She has also served in the Territorial Force Nursing Service.

